# Glycerophospholipids in Red Blood Cells Are Associated with Aerobic Performance in Young Swimmers

**DOI:** 10.3390/nu16060765

**Published:** 2024-03-07

**Authors:** Álex Aparecido Rosini Silva, Vanessa Bertolucci, Pedro Paulo Menezes Scariot, João Pedro da Cruz, Flavio Marcio Macedo Mendes, Danilo Cardoso de Oliveira, Catharina Delry Plumari, Ivan Gustavo Masseli Dos Reis, Andreia Melo Porcari, Leonardo Henrique Dalcheco Messias

**Affiliations:** 1 MS^4^Life Laboratory of Mass Spectrometry, Health Sciences Postgraduate Program, São Francisco University, Bragança Paulista 12916-900, SP, Brazil; alexrosinisilva@hotmail.com (Á.A.R.S.); flaviosistema@hotmail.com (F.M.M.M.); danilo.colivera@gmail.com (D.C.d.O.); catharinaplumari@gmail.com (C.D.P.); andreia.porcari@usf.edu.br (A.M.P.); 2 Research Group on Technology Applied to Exercise Physiology—GTAFE, Health Sciences Postgraduate Program, São Francisco University, Bragança Paulista 12916-900, SP, Brazil; vanessa.bertolucci@mail.usf.edu.br (V.B.); pedroppms@yahoo.com.br (P.P.M.S.); jpdacruz97@hotmail.com (J.P.d.C.); ivan.reis@usf.edu.br (I.G.M.D.R.)

**Keywords:** lipidomics, swimming, red blood cells, critical velocity, aerobic performance

## Abstract

This study aimed to characterize the composition of lipids in the red blood cells (RBCs) of adolescent swimmers and correlate this lipidome with the aerobic performance of the athletes. Five experimental assessments were performed by 37 adolescent swimmers. During the first session, the athletes went to the laboratory facility for venous blood sampling. The critical velocity protocol was conducted over the 4 subsequent days to measure aerobic performance (CV), comprising maximal efforts over distances of 100, 200, 400, and 800 m in a swimming pool. RBCs were obtained and extracted for analysis using the liquid chromatography—high resolution mass spectrometry untargeted approach. A total of 2146 ions were detected in the RBCs, of which 119 were identified. The enrichment pathway analysis indicated intermediary lipids in the glycerophospholipid, glycerolipid, sphingolipid, linoleic acid, and alpha-linolenic metabolisms, as well as pentose and glucuronate interconversions. A significant impact of the intermediary lipids was observed for the glycerophospholipid metabolism, including phosphatidylethanolamine (PE), phosphatidylcholine (PC), 1-acyl-sn-glycero-3-phosphocholine, sn-glycerol 3-phosphate, and phosphatidic acid. Inverse and significant associations were observed for PE 18:2/18:3 (r = −0.39; *p* = 0.015), PC 18:3/20:0 (r = −0.33; *p* = 0.041), and phosphatidic acid 18:0/0:0 (r = −0.47; *p* = 0.003) with aerobic performance. Swimmers who exhibited higher levels of aerobic performance also had the lowest abundance of PE, PC, and phosphatidic acid.

## 1. Introduction

Although biofluids are the most preferred biospecimen for biochemical analysis, a paucity of interest has been given exclusively to red blood cells (RBCs). The main role of RBCs is to act as carriers of both oxygen and carbon dioxide [1,2], explaining why these cells are so important for those interested in aerobic exercise performance [3,4]. The relevance of hemoglobin is widespread in scientific studies, but this is not the only aspect that needs to be considered in the transport of respiratory gases. Indeed, the membrane properties of RBCs are also able to affect their oxygen-carrying capacity [5]. Evidence supporting this comes from rheological studies showing that more deformable/flexible RBCs may move through the microcirculation more easily [6,7,8,9,10]. Among the different issues that may be explored regarding membrane components, one that deserves attention is the composition of lipids, which appears important in RBCs given the absence of nuclei and organelles [11,12,13,14].

Given the importance of lipid composition in RBCs, lipidomics appears as an important tool, not only for understanding the biophysics of RBCs but also for providing valuable insights into the determinants of aerobic exercise performance. Although the evaluation of lipids in sports is growing, research on the topic is still incipient. Only a few lipidomic studies have investigated RBCs in the context of physical exercise [15,16,17]. To the best of our knowledge, there is a lack of studies exploring whether lipids in RBCs are related to aerobic exercise performance. Liquid chromatography coupled to mass spectrometry (LC-MS)-based lipidomics has already been performed outside of the sports context [18,19,20]. Indeed, MS-based lipidomics has emerged as a promising source of information, with considerable sensitivity and the ability to detect thousands of metabolites simultaneously. Therefore, by using an untargeted lipidomic approach, the first aim of the current work was to characterize the composition of lipids in the RBCs of swimmers, which are known to have a high dependence on aerobic metabolism as an energy requirement [21]. The second and main goal of this research was to verify the possible relationships between RBC lipidome and aerobic performance. For this, we used eigenvector centrality to understand which lipids would be correlated with the critical velocity, a valid measure of aerobic capacity [22,23].

## 2. Materials and Methods

### 2.1. Participants

**Figure 1:**
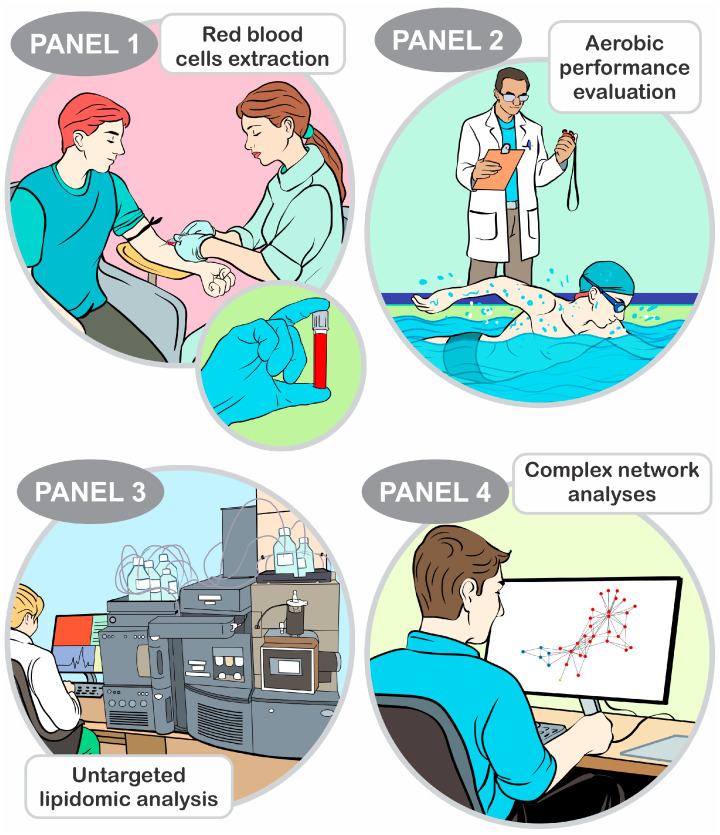
Experimental design of the study. Red blood cells (RBC) were collected by centrifugation of venous blood samples (Panel 1). The RBCs were preserved at −80 °C until further use. Subsequently, the swimmers underwent four predictive trials at 100, 200, 400, and 800 m to determine the critical velocity (Panel 2). Panel 3 shows that untargeted lipidomics were performed using an ultra-high-performance liquid chromatography system coupled to a quadrupole time-of-flight mass spectrometer. In Panel 4, the complex network and eigenvector centrality identified the lipids that were most relevant for the critical velocity, which was the target of this analysis.

Data from the participants included in this study were published before, but concerning distinct analysis [24]. Five experimental assessments were performed by 37 adolescent swimmers (male, n = 19; age = 15 ± 2 years; body mass = 61 ± 11 kg; height = 166 ± 16 cm; female, n = 18; age = 14 ± 2 years; body mass = 55 ± 9 kg; height = 160 ± 7 cm). The athletes went to the laboratory facility during the first session for venous blood sampling and anthropometric measurements. The critical velocity protocol was conducted over the 4 subsequent days (48 h apart at the same time of day) to measure aerobic performance, that is, the aerobic component of the critical velocity protocol. After the identification of molecules in the RBCs by the lipidomic procedure, the complex networks elicited the lipids with higher relevance for aerobic performance (Figure 1).

According to the training periodization created by coaches, the swimmers were at the start of the general preparation period. Coaches were advised by researchers to not conduct physical training during the experimental time interval. Hence, during the critical velocity protocol, athletes only engaged in light, leisurely activities. Throughout the experiment, researchers instructed athletes to keep the same individual hydration/food habits.

### 2.2. Determination of Aerobic Performance

The critical velocity protocol was conducted on four randomized maximal efforts over distances of 100, 200, 400, and 800 m in a swimming pool (25 m). Athletes were encouraged by researchers and coaches to provide their best efforts during trials. For determination of the aerobic estimate (e.g., CV), the equation D = CV × t + AWC was applied, where D is equivalent to distance, t is related to time to cover the distance, AWC (e.g., anaerobic work capacity) refers to the y-intercept, and CV relates to the slope of the regression. Given the purposes of this report, only CV was considered for the relationship with the RBCs’ lipids.

### 2.3. Red Blood Cell Profiles

Before having their blood drawn, adolescent swimmers were instructed to abstain from alcohol and unusual foods and drinks for 3 days. A volume of 5 mL of venous blood was drawn by a skilled nurse for hematological evaluations. Samples were brought to a specialized laboratory facility where the Coulter LH 750 hematology analyzer (Beckman Coulter, Miami, FL, USA) [25] evaluated the red blood cell profile, including red blood cell count, hemoglobin, hematocrit, mean corpuscular volume, mean corpuscular hemoglobin, mean corpuscular hemoglobin concentration, and red cell distribution.

### 2.4. Extraction and Lipidomic Analysis of RBCs

In addition to the blood sample collected for hematological analysis, another 5 mL of venous blood was collected for lipidomic analysis in a separate tube. The sample was centrifuged at 1500 rpm for 10 min, allowing the separation of plasma and RBC. The RBC samples were transferred to other tubes and frozen at −80 °C until extraction. The extraction was based on Gil, et al. [26]. Each sample (200 μL) was then extracted by adding 850 μL of a cold solution composed of methanol (MeOH)/methyl-terc-butyl-ether (MTBE)/chloroform (CHCl_3_) (1.33:1:1, *v*/*v*/*v*). Afterwards, the samples were homogenized by vortexing for 30 min (2000 RPM at 22 °C), and then vortexed for an additional 30 s. Samples were again centrifuged (13,000 RPM, 10 min, 4 °C), and the supernatant was collected and dried over a nitrogen gas (N2) flow. Samples were resuspended in 200 μL of a solution of isopropanol (ISP)/acetonitrile (ACN)/H_2_O (2:1:1 *v*/*v*/*v*).

A total of 25 μL of each resuspended sample was collected to compose a pooled sample used as quality control (QC). To check deviations in extraction and system stability, QC samples were injected after 10 samples. Furthermore, a QC sample was used at the beginning of the experiment to perform instrumental stabilization of the LC-MS system. Participant samples were extracted and analyzed randomly to observe biological variation and minimize instrumental bias.

The analyses were adapted from Silva et al. [27]. An ACQUITY UPLC was used, coupled to a XEVO-G2XS (QToF) quadruple time-of-flight mass spectrometer (Waters, Manchester, UK) equipped with an electrospray ionization (ESI) source operated in the negative ionization mode. For lipidomic analysis, we employed an ACQUITY UPLC^®^ CSH C18 column (2.1 mm × 100 mm × 1.7 μm, Waters), using the mobile phase A composed of an ACN:H_2_O solution (60:40, *v*/*v*) with 10 mM ammonium formate + 0.1% formic acid, and the mobile phase B, composed of ISP/ACN (90:10, *v*/*v*) with 10 mM ammonium formate + 0.1% formic acid. The flow rate was 0.4 mL min^−1^. Initially, the column was conditioned with 40% B, increasing to 43% over the next 2 min and subsequently to 50% within 0.1 min. In the next 9.9 min, the gradient was gradually increased to 54% B and then to 70% B in 0.1 min. At the end of the gradient, B was increased to 99% over 5.9 min; after this period, solution B returned to 40% in 0.1 min, balancing the column for the next injection for the next 1.9 min.

The mass spectrometer was operated in MS^E^ mode with an *m*/*z* range of 50–1200 Da, and an acquisition time of 0.5 s per scan. MS^E^ analysis was operated at 6 V for low-collision energy and a ramp of 20–50 V for high collision energy. Leucine enkephalin (molecular weight = 555.62; 200 pg L^−1^ in 1:1 ACN: H_2_O, *v*/*v*) was used as the lock mass for mass accuracy, and a 0.5 mM sodium formate solution was used for calibration. Other parameters were as follows: source temperature = 140 °C, desolvation temperature = 550 °C, desolvation gas flow = 900 L h^−1^, capillary voltage = 2.5 kV, and cone voltage = 40 V.

### 2.5. Data Processing and Putative Identification of Metabolites

The LC-MS raw files were processed using the Progenesis™ QI software v2.4 (Non-linear Dynamics, Newcastle, UK), which allowed the selection of possible adducts, peak alignment, deconvolution, and annotation of compounds based on MS^E^ experiments. An alignment score of 95% was adopted. The adducts [M+H]^+^, [M+K]^+^, [M+Na]^+^, [M+ACN+H]^+^, [M+H−H_2_O]^+^ and [M+NH_4_]^+^ were considered for the positive acquisition mode, and [M−H]^−^, [M+Cl]^−^, [M−H_2_O−H]^−^, and [M+FA−H]^−^ for the negative acquisition mode. Progenesis QI generates an intensity table of the features, which are the ions of each sample, labeled according to their nominal masses and retention times, as a function of their intensity, considered as the areas of the extracted ion chromatogram.

Due to low- and high-energy acquisition enabled by the use of MS^E^, we have information on precursor ions (low energy) and fragments (high energy) in the same spectrum. A precursor mass error of ≤5 ppm was considered, and a fragment tolerance of ≤10 ppm. Fragmentation profile, mass accuracy, mass error, and isotope similarity were evaluated to accept the annotated molecules. To allow the compatibility of Progenesis PQI data and external SDF-based spectra libraries, we used an in-house software named “SDF2PQI” to increase the number of fragment matches [28]. SDF2PQI was recently detailed elsewhere and is available free of charge. External SDF-based spectra libraries were used, such as LipidMaps (http://www.lipidmaps.org/, accessed on 7 December 2023), the Human Metabolome Database (http://www.hmdb.ca/metabolites), and the MoNA—MassBank of North America (https://mona.fiehnlab.ucdavis.edu/).

### 2.6. Statistical Analyses

The complex network was created based on only significant (*p* < 0.05) correlations [24,29] between CV and erythrocyte lipids, regardless of the correlation coefficient. In the network, each variable that was associated with another was represented as a node, and the associations between the variables’ edges connecting these nodes represented connections between nodes. By selecting CV as a target inside the topology, weighted and targeted complex networks were developed. These methods gave positive weights to both positive and inverse correlations equally, regardless of the correlation’s direction.

In the eigenvector approach, the centrality of a node is calculated using the target techniques based on the centrality of its neighbors and the weights of its edge connections. The edge weights were determined by multiplying Pearson’s correlation coefficient between the nodes connected by the edge (which can vary from 0.01 to 1; higher means closer) by the edge’s degree of closeness to the target node CV (which can vary from 0.01 to 1; higher is better). The centrality eigenvector values were acquired using a Python (version 3.9.3) application created specifically for the study and the NetworkX 2.5 package [30]. The Shapiro–Wilk test confirmed the data normality, and the Pearson approach was used for the correlation analysis. Data are expressed as mean ± standard deviation.

To select features based on the eigenvector approach, a threshold of ≥0.0001 was adopted. The principal component analysis (PCA) and enrichment analysis were performed using MetaboAnalyst 5.0 software. The relative standard deviation (RSD) was calculated for the intra-batch QC group, and those features found with an RSD < 30% were not considered for statistical analysis. The dataset was sum-normalized, log-transformed, and scaled by pareto. The false discovery rate (FDR) was applied to control the rate of false positive findings, considering *p* < 0.05. The impact in enrichment analysis refers to a quantitative measure that assesses the biological relevance or importance of enriched pathways.

## 3. Results

Performances on the 100, 200, 400, and 800 m were obtained at 73 ± 9 s, 170 ± 16 s, 354 ± 43 s, and 747 ± 84 s, respectively. Consequently, the CV was calculated at 1.05 ± 0.11 m/s, with a high R2 (0.999 ± 0.001). Parallel to the mass spectrometry analysis, the red blood cell profile was determined (red blood cell count = 4.83 ± 0.37 10^6^/μL; hemoglobin = 13.8 ± 1.01 g/dL; hematocrit = 42.8 ± 2.95%; mean corpuscular volume = 88.6 ± 3.40 fl; mean corpuscular hemoglobin = 28.7 ± 1.04 pg; mean corpuscular hemoglobin concentration = 32.3 ± 0.5 10^6^/μL; red blood cell distribution = 13.4 ± 0.4%).

LC-MS raw data were processed, and 2823 signals were detected and filtered by RSD for the QC group. The analytical quality and the LC-MS reproducibility can be observed by the clustering of the QC group in PCA (Supplementary Figure S1). The selection of features was based on eigenvector value (≥0.0001), with 266 indicated as the differential, identified (n = 119), and grouped by chemical subclass.

Table 1 presents the total of erythrocyte lipids detected, identified, and relevant for the CV of adolescent swimmers. Lipids from the glycerophospholipids and sphingolipids classes comprised 81.5% of the identified ones. The complete list of lipids, main classes, sub-classes, fragments, and the respective eigenvector values can be visualized in Supplementary Table S1.

**Figure 2:**
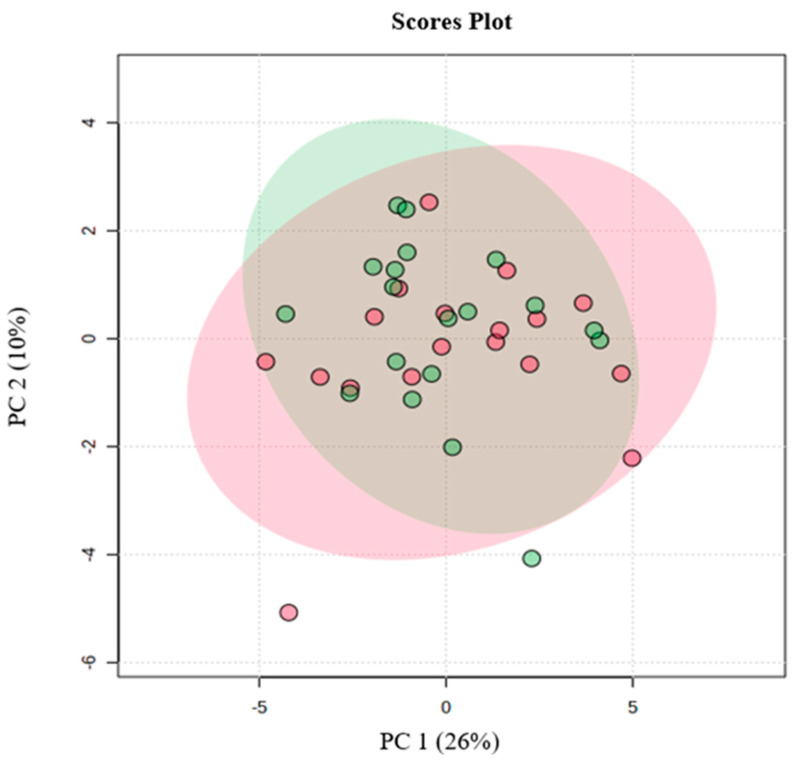
The principal component analysis (PCA) between male and female adolescent swimmers regarding the lipids identified in lipidomic analysis that were relevant for aerobic performance; red—female; green—male.

Before proceeding with further analysis on the lipids relevant for aerobic performance, a comparison regarding the identified molecules between male and female athletes was performed. The PCA analysis showed that the data variability was not sufficient to discriminate between male and female adolescent swimmers by the lipids (Figure 2). Based on this result, the enrichment and the last analysis considered the total sample regardless of sex.

**Figure 3:**
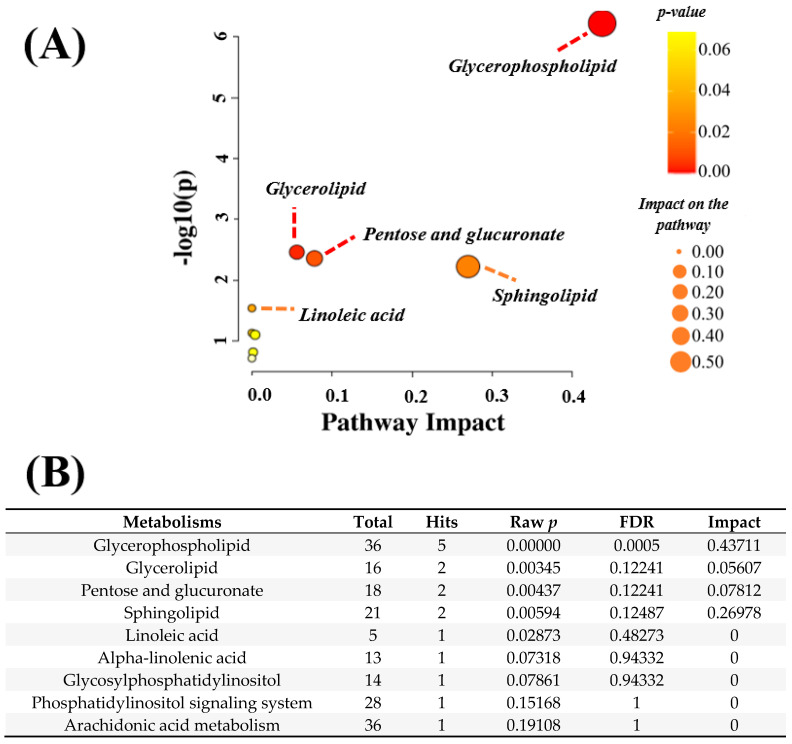
The enrichment pathway analysis (**A**) is based on the erythrocyte lipids of adolescent swimmers and the statistics of each highlighted metabolism (**B**); FDR—false discovery rate; *p* < 0.05.

The enrichment pathway analysis was performed with all metabolites (n = 119), which indicated intermediary lipids in the glycerophospholipid, glycerolipid, sphingolipid, linoleic acid, and alpha-linolenic metabolisms, as well as pentose and glucuronate interconversions (Figure 3). A significant impact of the intermediary lipids was observed for the glycerophospholipid metabolism, including phosphatidylethanolamines (PEs), phosphatidylcholines (PCs), 1-acyl-sn-glycero-3-phosphocholine, sn-glycerol 3-phosphate, and phosphatidic acids. While the latter two classes of lipids also have intermediate glycerolipid metabolisms, sphingomyelin (SM) and ceramides (CERs) were found to be hits of the sphingolipid metabolism. In addition to glycerophospholipid metabolism, PC also has intermediate linoleic acid metabolism, which was highlighted in this pathway. Lastly, D-xylose and D-xylonolactone were the hits in pentose and glucuronate interconversions. The entire glycerophospholipid metabolism is presented in Supplementary Figure S2.

**Figure 4:**
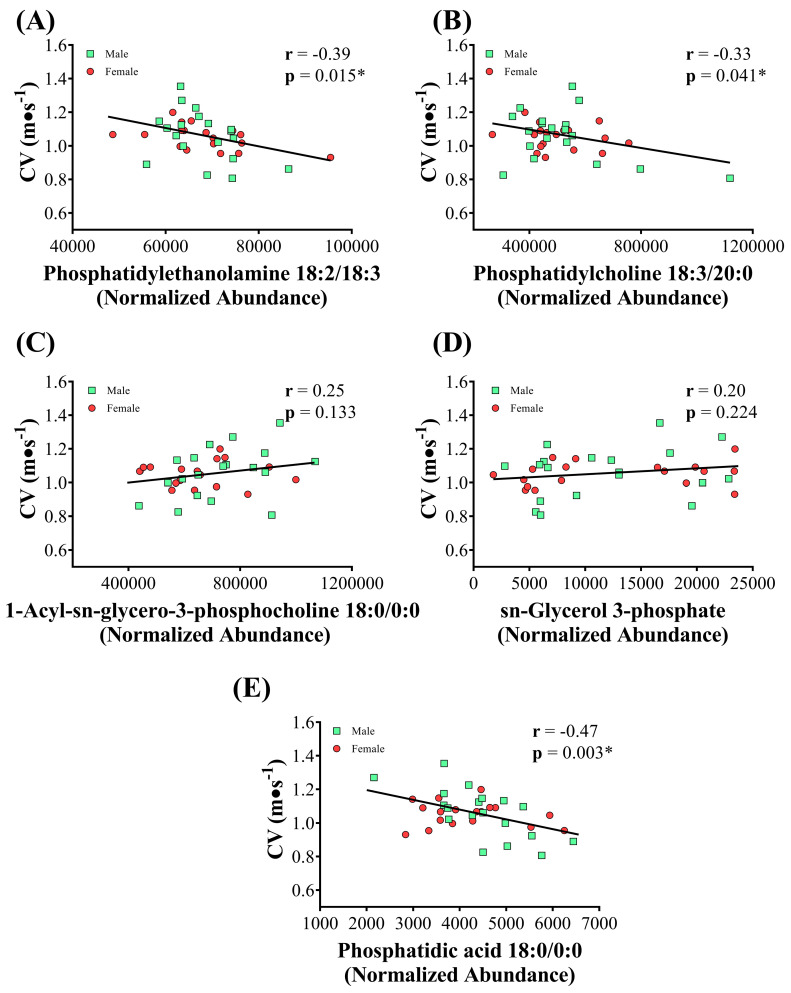
Correlation between the RBC lipids highlighted in the enrichment analysis for the glycerophospholipid metabolism pathway with the critical velocity (CV) of adolescent swimmers. (**A**) correlation between phosphatidylethanolamine 18:2/18:3 with CV; (**B**) correlation between phosphatidylcholine 18:3/20:0 with CV; (**C**) correlation between 1-acyl-sn-glycero-3-phosphocholine 18:0/0:0 with CV; (**D**) correlation between sn-glycerol 3-phosphate with CV; (**E**) correlation between phosphatidic acid 18:0/0:0 with CV. Red—female; green—male; * *p* < 0.05.

The lipids highlighted in the glycerophospholipid metabolism pathway were correlated with the CV (Figure 4). Inverse and significant associations were observed for PE 18:2/18:3 (Figure 4A), PC 18:3/20:0 (Figure 4B), and phosphatidic acid 18:0/0:0 (Figure 4E) with the CV.

## 4. Discussion

The lipidomics approach, along with the targeted network analysis, revealed 119 RBC metabolites associated with the CV of adolescent swimmers. The differential abundance of lipids resulted in a significant impact on glycerophospholipid metabolism. Among the glycerophospholipids, compounds representing subclasses such as phosphatidylethanolamine, phosphatidylcholine, and phosphatidic acid were inversely correlated with critical velocity. The data presented here can support the view that these glycerophospholipids are downregulated in athletes with high aerobic performance.

In our enrichment pathway analysis, we observed a marked relevance of glycerophospholipid metabolism (Figure 3A). Since glycerophospholipids are a class of lipids that constitute a major component of cell membranes [31], they are expected to have an important role for RBCs. Our finding, together with those of others reported in the literature [14,16,32], reinforces the idea that glycerophospholipids are inexorably linked to the structure of RBCs. The reason why the metabolism of glycerophospholipid was highlighted in the RBCs of swimmers is an interesting finding and deserves further consideration. An enhanced use of fat metabolism can be responsible for the prominence of glycerophospholipid metabolism. Thus, we hypothesize that aerobic activities included in the scope of swimming training may play a role in stimulating fat-oxidative pathways. The results in the present study are not sufficient to demonstrate a causal effect of aerobic training on glycerophospholipids, but it would not be surprising to find an increased flux of lipids in the circulation of endurance-trained swimmers. Given that RBCs are incapable of performing biosynthesis due to the lack of organelles, it is opportune to mention that the membrane lipid composition of RBCs depends on the exchange with the plasma lipids [12,13,14]. In support of this, hemorheological changes have been demonstrated in rabbits with hypercholesterolemia [33]. Other researchers have proved that RBCs are sensitive to plasma lipids [34,35,36].

With regard to the correlations, we found a significant inverse relationship between CV and three metabolites of glycerophospholipid metabolism. Swimmers who exhibited higher levels of CV (a measure related to aerobic performance) also had the lowest abundance of phosphatidylethanolamine at 18:2/18:3 (r = −0.39; *p* = 0.015), phosphatidylcholine at 18:3/20:0 (r = −0.33; *p* = 0.041), and phosphatidic acid at 18:0/0:0 (r = −0.47; *p*= 0.003). At the moment, we have no conditions to elucidate these relationships, but it appears appropriate to speculate that changes in RBC glycerophospholipids may be indirectly linked with physical fitness. Phosphatidylethanolamine is a key regulator that we can use as an example. There are reasons to believe that a high amount of phosphatidylethanolamine is indicative of poor health. Supporting this, increased phosphatidylethanolamine levels have been found in non-alcoholic steatohepatitis patients [37]. An increased phosphatidylethanolamine content of platelets has also been found in patients with poorly controlled diabetes [38], and increased phosphatidylethanolamine has been described in cancer [39] and hypertension [40].

**Figure 5:**
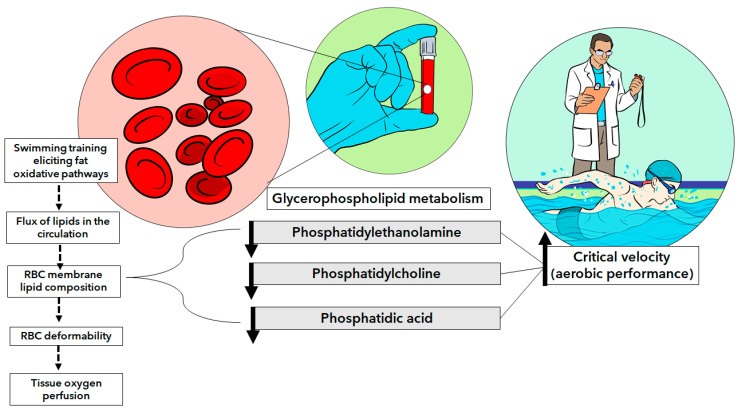
The main findings and possible interpretations (dashed arrows) of the study highlighting the glycerophospholipids subclasses in red blood cells that were inversely correlated with critical velocity (a measure of aerobic performance).

There is a growing body of evidence that supports the idea that phosphatidylethanolamines can be modified by glycation, oxidation, and other chemical processes [41,42,43,44,45]. With this in mind, it is reasonable to expect harmful changes in membrane thickness and rigidity, and thus RBC locomotion could be seriously compromised [46,47]. This agrees with previous findings confirming that acute exercise decreases RBC deformability [48,49,50,51]. In contrast to acute exercise-related alterations in RBCs, exercise training is known to improve hemorheological parameters [50]. Although we have no measurements of hemorheological parameters (e.g., whole-blood viscosity, RBC aggregation, RBC deformability), we believe that endurance-trained swimmers have enhanced blood flow capacity (likely facilitating oxygen diffusion and tissue repair during rest moments). This, in turn, could be achieved with highly deformable RBCs, which would have conditions to pass (due to reduced membrane stiffness) through the smallest capillaries. All these findings are intriguing and emphasize that the RBC has a complex role, which further depends on the condition in which these cells were harvested, such as exercise or rest (which was our case). Future in-depth studies will need to be conducted to understand mechanisms involving aerobic capacity and RBC glycerophospholipids, comparing both rest and exercise conditions from an integrated perspective. Some of the main findings are sketched in Figure 5.

There are some studies employing metabolomics in the context of swimming [52,53,54]. Researchers have characterized the serum metabolic profile of swimmers to identify fitness levels or predict competitive potential. Cai et al. [55] discriminated swimmers with different competitive levels using high-density lipoprotein, glutamine, methanol, and α-glucose. On the other hand, plasma tyrosine was inversely associated with aerobic performance [56]. Although the presented knowledge has contributed to the characterization of different metabolites present in the plasma and serum of swimmers, there is a scarcity of studies exploring RBCs. The novelty of our study was to analyze the RBC lipidome in athletes under rest conditions. The researchers employing lipidomics in RBCs have focused on the metabolic changes that occur after an exercise bout [15,16]. Knowing all the benefits that RBCs can bring to the aerobic physical performance of swimmers and the scientific potential of the lipidomic approach, it becomes of great value to characterize the lipid molecules present in the RBCs of young swimming athletes.

Our study has some limitations. We did not examine whether athletes would have a different RBC lipidome under exercise or diet situations. Although linked to aerobic exercise performance, we did not analyze the shape of RBCs by histological assays. Given that RBC membrane fluidity and lipid composition may be affected by nutrition factors [57,58,59,60,61,62], it is imperative to account for this factor in a further experimental design. Therefore, more studies are needed to get a better understanding of the impact of athletes’ diets on the RBC membrane lipidome. At the moment, we do not have enough information on food ingested by swimmers, despite the fasting state of athletes. Despite limitations, our study is a first step in exploring RBC lipidomics in adolescent swimmers. To our knowledge, our study is the first to demonstrate a connection between some glycerophospholipids in RBCs and aerobic exercise performance (this knowledge could be useful in the fields of medicine and biology). Another approach to be highlighted is the use of critical velocity protocol, which can access aerobic capacity without the necessity of blood collection. Still, we investigated whether the strength and direction of the association were dependent on sex. The PCA analysis found no difference between male and female adolescent swimmers by the lipids, and the dispersion was homogeneous without any agglomeration for females (red circle) and males (green square). This supports the possibility that other factors beyond gender are responsible for the changes in RBC glycerophospholipids in athletes. We believe that glycerophospholipids in RBCs are an important link between aerobic exercise performance and the regulation of oxygen-carrying capacity. Considering that RBCs regulate several processes that are pivotal in physiology, our findings provide new insights and bring us much closer to understanding RBC adaptations in athletes with different levels of aerobic capacity. The next step will be to explore whether adaptations resulting from aerobic training could be attributed to changes in the RBC lipidome.

## 5. Conclusions

A significant impact of the intermediary lipids was observed for glycerophospholipid metabolism in the red blood cells of young swimmers. Among these lipids, phosphatidylethanolamine 18:2/18:3, phosphatidylcholine 18:3/20:0, and phosphatidic acid 18:0/0:0 were inversely correlated with CV, suggesting that these are downregulated in athletes with high aerobic performance.

## Figures and Tables

**Table 1:** Lipids that were detected and identified in RBCs and also relevant (i.e., eigenvector) for the aerobic performance of adolescent swimmers.

	n	Eigenvector Range (A.U)
**Total Features with RSD < 30%**	2146	0–0.088345
**Features selected by eigenvector**	266	≥0.0001
**Lipids Identified**	119	0.000102–0.088345
*Glycerophospholipids*	65	0.000102–0.085850
*Sphingolipids*	32	0.000197–0.088324
*Fatty Acyls*	6	0.000104–0.088345
*Neutral glycosphingolipids*	3	0.000174–0.068406
*Glycerolipids*	2	0.001768–0.074106
Others classes *	11	0.000112–0.079785

A.U—arbitrary units; * Others include carboxylic acids and derivatives, organooxygen compounds, lactones, phenols, pyrans, pyridines and derivatives, and dihydrofurans.

## Data Availability

Data are contained within the article and Supplementary Materials.

## Supplementary Materials

The following supporting information can be downloaded at: https://www.mdpi.com/article/10.3390/nu16060765/s1, Figure S1: Principal Component Analysis (PCA) results. PCA using all metabolites detected. The red point represents the QC Samples, and the green point represents Swimmers Samples; Figure S2: The glycerophospholipid metabolism pathway and the lipids (i.e., red) that were identified in erythrocytes of young swimmers; Table S1: Compounds identified (n = 119) for red blood cell lipidomics.
