# Supplementary material for: Glycerophospholipids in Red Blood Cells Are Associated with Aerobic Performance in Young Swimmers
Source: Nutrients. 2024 Mar 7;16(6):765. doi: 10.3390/nu16060765 (PMC10974386; doi:10.3390/nu16060765)
Supplement: Supplementary file 1 [file nutrients-16-00765-s001.zip › nutrients-2869455-supplementary.pdf]

Supplementary Files

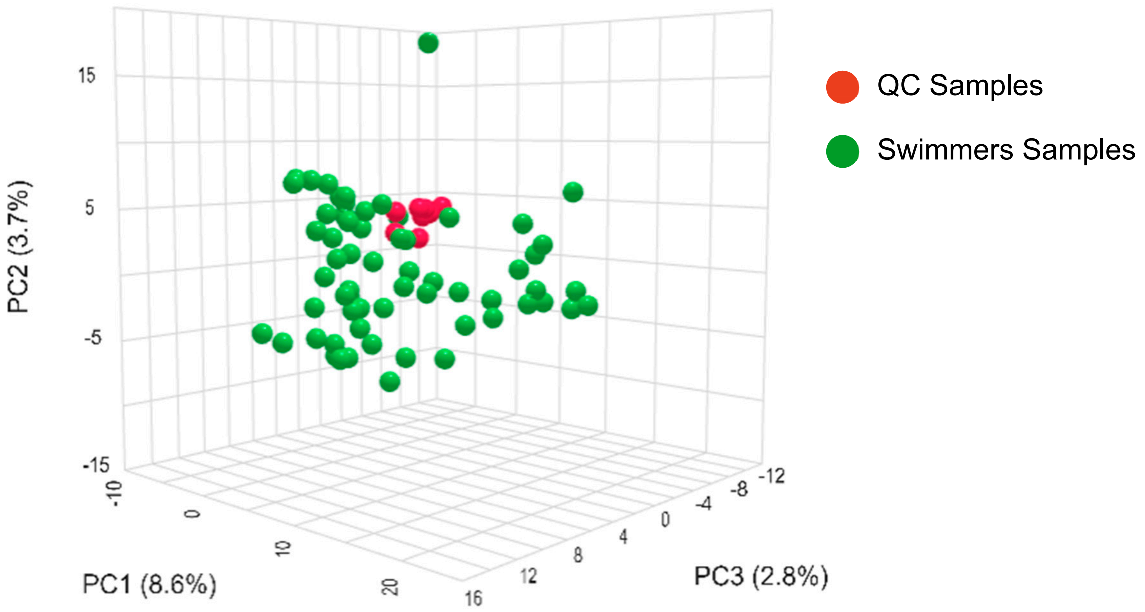

Supplementary Figure S1. Principal Component Analysis (PCA) results. PCA using all metabolites detected. The red point represents the QC Samples, and the green point represents Swimmers Samples.

Glycerophospholipid metabolism

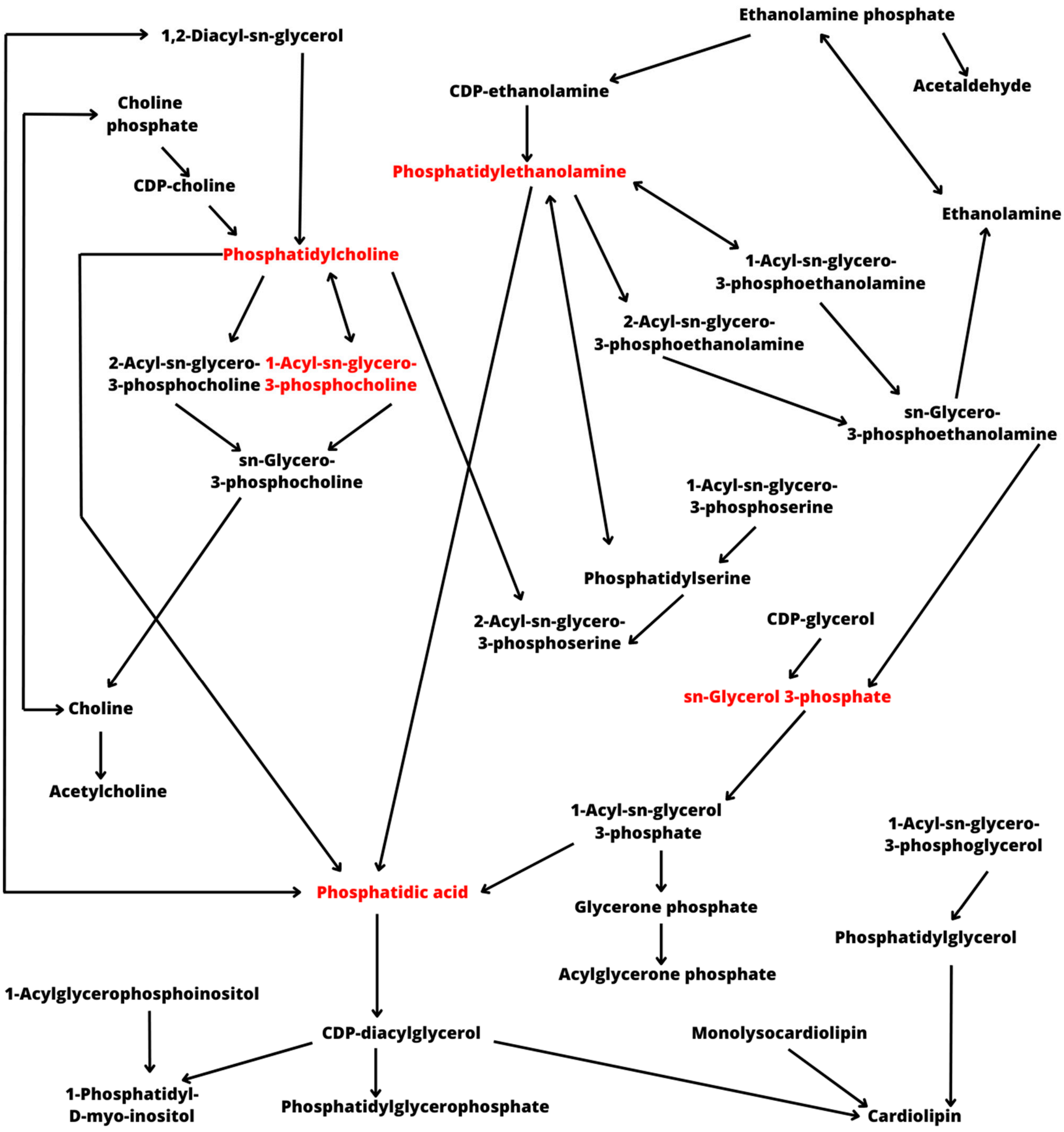

Supplementary Figure S2. The glycerophospholipid metabolism pathway and the lipids (i.e red) that were identified in erythrocytes of young swimmers.

Supplementary Table S1. Compounds identified (n=119) for red blood cell lipidomics.

m/z measured	Retention Time (min)	Molecular Formula	Adduct	Compound Name	Mass Error (*ppm*)	Eigenvector	Main Class	SubClass	Matched Fragments
217,0828	0,28	$C_7H_{12}N_2O_3$	M+FA-H	Glycylproline	-1,39	0,064770	Carboxylic acids and derivatives	Amino acids, peptides, and analogues	188.0414; 170.0301; 157.0977; 128.0348; 114.0554; 87.0080
226,0728	0,28	$C_9H_{11}NO_3$	M+FA-H	4-HOPC4a	3,84	0,002794	Pyridines and derivatives	#N/D	135.0307; 107.0362; 85.0289
147,0293	0,30	$C_5H_8O_5$	M-H	Xylonolactone	-4,88	0,058714	Lactones	Delta valerolactones	135.0306; 134.0220; 115.0395; 89.0237; 87.0080; 85.0290
135,0306	0,31	$C_4H_8O_5$	M-H	Erythronic acid	4,81	0,055722	Organooxygen compounds	Carbohydrates and carbohydrate conjugates	134.0231; 129.0190; 113.0239; 101.0239; 89.0237; 87.0080
149,0453	0,31	$C_5H_{10}O_5$	M-H	Xylose	-1,92	0,002283	Organooxygen compounds	Carbohydrates and carbohydrate conjugates	135.0306; 117.0550; 115.0397; 103.0395; 89.0237; 87.0081
117,0552	0,33	$C_5H_{10}O_3$	M-H	β-hydroxyvaleric acid	-4,14	0,003026	Fatty Acyls	Fatty acids and conjugates	103.0395; 99.0446; 89.0237; 83.0494; 81.0346; 71.0127
151,0395	0,33	$C_8H_{10}O_4$	M-H_2O-H	Furaneol acetate	-3,52	0,002740	Dihydrofurans	Furanones	144.0451; 135.0482; 115.0400; 108.0217; 97.0288; 92.0249
157,0491	0,33	$C_7H_{10}O_4$	M-H	xi-2,3-Dihydro-3,5-dihydroxy-6-methyl-4H-pyran-4-one	-1,17	0,000112	Pyrans	Pyranones and derivatives	144.0451; 143.0713; 139.0401; 130.0635; 92.0249; 89.0237
193,0496	0,33	$C_{10}H_{12}O_5$	M-H_2O-H	Oresbiusin A	-4,82	0,060215	Phenols	Benzenediols	157.0326; 154.0681; 123.0447; 115.0203; 108.0203; 103.0400
217,0117	0,33	$C_3H_9O_6P$	M+FA-H	Glycerol 3-phosphate	-1,26	0,003992	Glycerophospholipids	Glycerophosphates	152.9953; 135.0307; 103.0395; 96.9691; 89.0237; 87.0081
145,0866	0,35	$C_7H_{14}O_3$	M-H	Methyl DL-Leucate	-2,57	0,000104	Fatty Acyls	Fatty acid esters	143.0714; 135.0307; 103.0394; 89.0237
518,2878	0,54	$C_{27}H_{41}N_3O_7$	M-H	Vignatic acid B	1,26	0,060259	Carboxylic acids and derivatives	Amino acids, peptides, and analogues	441.2524; 329.1893; 327.2321
341,2323	0,59	$C_{18}H_{32}O_3$	M+FA-H	FA 18:2;O	-3,39	0,052723	Fatty Acyls	Fatty Acids and Conjugates	281.2472; 279.2319; 255.2321; 173.0820; 129.0554; 109.0292
450,2621	0,59	$C_{21}H_{42}NO_7P$	M-H	LPE 16:1/0:0	-1,12	0,002725	Glycerophospholipids	Glycerophosphoethanolamines	297.2427; 295.2271; 279.2319; 253.2167; 214.0481; 184.0161
500,2773	0,59	$C_{25}H_{44}NO_7P$	M-H	LPE 20:4/0:0	-1,94	0,002348	Glycerophospholipids	Glycerophosphoethanolamines	303.2318; 214.0481
480,3082	0,75	$C_{23}H_{48}NO_7P$	M-H	LPE 18:0/0:0	-2,74	0,058921	Glycerophospholipids	Glycerophosphoethanolamines	452.2776; 281.2476; 196.0637; 140.0113
481,2574	0,75	$C_{21}H_{41}O_7P$	M+FA-H	LPA 18:1/0:0	0,58	0,004145	Glycerophospholipids	Glycerophosphates	437.2676; 281.2476; 255.2320; 152.9952
540,3301	0,75	$C_{24}H_{50}NO_7P$	M+FA-H	LPC 16:0/0:0	-1,26	0,063434	Glycerophospholipids	Glycerophosphocholines	540.3305; 255.2320; 224.0685
506,3249	0,77	$C_{25}H_{50}NO_7P$	M-H	LPE 20:1	-0,53	0,004318	Glycerophospholipids	Glycerophosphoethanolamines	478.2932; 196.0373
524,3367	0,84	$C_{24}H_{50}NO_6P$	M+FA-H	LPC O-16:1/0:0	1,93	0,076892	Glycerophospholipids	Glycerophosphocholines	464.3136; 436.2824; 403.2606; 269.2476; 242.0795; 225.0742
436,2825	0,89	$C_{20}H_{41}NO_4S$	M+FA-H	N-Stearoyl Taurine	-2,41	0,004063	Carboxylic acids and derivatives	Amino acids, peptides, and analogues	269.2477
466,3286	0,89	$C_{24}H_{43}N_3O_3$	M+FA-H	N-Stearoyl Histidine	-0,04	0,079785	Carboxylic acids and derivatives	Amino acids, peptides, and analogues	436.2824; 283.2633; 269.2477; 242.0791; 224.0684

526,3514	0,89	$C_{24}H_{52}NO_6P$	M+FA-H	LPC O-16:0/0:0	-0,02	0,085850	Glycerophospholipids	Glycerophosphocholines	494.3244; 452.2777; 269.2477; 342.0791
599,3224	0,89	$C_{27}H_{53}O_{12}P$	M-H	LPI 18:0/0:0	3,68	0,000112	Glycerophospholipids	Glycerophosphoinositols	437.2676; 419.2558; 283.2633; 101.0238
524,2991	0,91	$C_{24}H_{48}NO_9P$	M-H	LPS 18:0	-0,58	0,000205	Glycerophospholipids	Glycerophosphoserines	437.2676; 419.2558; 152.9952
492,3441	0,93	$C_{25}H_{52}NO_6P$	M-H	LPE O-20:0	-3,82	0,055799	Glycerophospholipids	Glycerophosphoethanolamines	462.2986; 436.2833; 140.0113
466,2960	0,94	$C_{22}H_{46}NO_7P$	M-H	LPC 14:0/0:0	4,40	0,004391	Glycerophospholipids	Glycerophosphocholines	452.2776; 436.2823; 271.2276
552,3096	0,96	$C_{29}H_{48}NO_7P$	M-H	LPE 24:6	0,04	0,000104	Glycerophospholipids	Glycerophosphoethanolamines	535.3047; 152.9952; 122.0015
506,3251	1,10	$C_{25}H_{50}NO_7P$	M-H	LPE 20:1	-0,19	0,058793	Glycerophospholipids	Glycerophosphoethanolamines	464.3137
568,3617	1,10	$C_{26}H_{54}NO_7P$	M+FA-H	LPC 18:0/0:0	-0,64	0,000109	Glycerophospholipids	Glycerophosphocholines	508.3401; 283.2632; 224.0687
480,3088	1,16	$C_{23}H_{48}NO_7P$	M-H	LPE 18:0/0:0	-1,56	0,003046	Glycerophospholipids	Glycerophosphoethanolamines	465.3179;446.3041;309.2797; 196.0374
872,5237	1,37	$C_{50}H_{78}NO_8P$	M+Na-2H	PE-NMe(44:11)	3,02	0,002887	Glycerophospholipids	Glycerophosphoethanolamines	856.5167; 854.5194; 808.4825; 169.0473
817,6420	10,81	$C_{44}H_{89}N_2O_6P$	M+FA-H	SM 39:1;O_2	-2,62	0,003444	Sphingolipids	Phosphosphingolipids	757.6235
778,5774	11,53	$C_{45}H_{84}NO_8P$	M-H_2O-H	PE 22:2/18:1	2,27	0,002040	Glycerophospholipids	Glycerophosphoethanolamines	281.2477; 140.0110
728,5607	11,59	$C_{41}H_{80}NO_7P$	M-H	PE P-18:1/18:0	0,98	0,004226	Glycerophospholipids	Glycerophosphoethanolamines	464.3143; 446.3037; 281.2476; 140.0113
664,5891	12,04	$C_{40}H_{77}NO_3$	M+FA-H	Cer 40:2;O_2	0,84	0,077881	Sphingolipids	Ceramides	618.5838
874,6683	12,14	$C_{52}H_{96}NO_8P$	M-H_2O-H	PE-NMe 46:4	-1,34	0,000117	Glycerophospholipids	Glycerophosphoethanolamines	857.6767; 168.0424
690,6046	12,43	$C_{42}H_{79}NO_3$	M+FA-H	Cer 42:3;O_2	0,67	0,072929	Sphingolipids	Ceramides	644.5989; 614.5878; 388.3576; 363.3286; 362.3428
664,5890	12,57	$C_{40}H_{77}NO_3$	M+FA-H	Cer 18:1;O_2/22:1	0,65	0,069419	Sphingolipids	Ceramides	620.5908; 618.5837; 588.5732; 339.3293; 321.3130
716,6206	12,72	$C_{44}H_{81}NO_3$	M+FA-H	Cer 44:4;O_2	1,17	0,074668	Sphingolipids	Ceramides	670.6161
678,6047	13,26	$C_{41}H_{79}NO_3$	M+FA-H	Cer 41:2;O_2	0,85	0,088324	Sphingolipids	Ceramides	632.5991
695,5971	13,26	$C_{46}H_{80}O_4$	M-H	FAHFA 46:5	-1,91	0,088345	Fatty Acyls	Fatty acids and conjugates	331.2635
710,6306	13,26	$C_{42}H_{83}NO_4$	M+FA-H	Cer 42:1;O_2	0,30	0,000304	Sphingolipids	Ceramides	664.6260; 646.6148; 632.5991
684,6144	13,33	$C_{40}H_{81}NO_4$	M+FA-H	Cer 40:0;O_2	-0,52	0,005597	Sphingolipids	Ceramides	638.6093
1221,8239	13,54	$C_{30}H_{62}NO_9P$	2M-H	LPS O-24:0;O	-1,02	0,068636	Glycerophospholipids	Glycerophosphoserines	1175.8191; 1134.7914
728,6415	13,54	$C_{42}H_{85}NO_5$	M+FA-H	Cer 42:0;O_3	0,78	0,000197	Sphingolipids	Ceramides	682.6255
662,6100	13,60	$C_{42}H_{81}NO_4$	M-H	HexCer 36:1;O	1,07	0,068406	Neutral glycosphingolipids	Simple Glc series	646.6144; 406.3690; 390.3729; 364.3577; 347.3312; 263.2374
692,6202	13,60	$C_{42}H_{81}NO_3$	M+FA-H	Cer 18:1;O2/24:1	0,52	0,083209	Sphingolipids	Ceramides	646.6144; 616.6036; 406.3681; 390.3729; 364.3577
709,6114	13,60	$C_{47}H_{84}O_5$	M-H_2O-H	DG 24:0/20:4	-3,58	0,074106	Glycerolipids	Diradylglycerols	692.6202; 347.3312

666,6042	13,65	$C_{40}H_{79}NO_3$	M+FA-H	Cer 40:1;O_2	0,03	0,055969	Sphingolipids	Ceramides	620.5988
799,6708	13,67	$C_{46}H_{93}N_2O_6P$	M-H	SM 17:1;O_2/24:0	1,16	0,004501	Sphingolipids	Phosphosphingolipids	366.3736; 349.3468
859,6918	13,67	$C_{47}H_{95}N_2O_6P$	M+FA-H	SM 18:1;O_2/24:0	0,97	0,000235	Sphingolipids	Phosphosphingolipids	1674.3823; 859.6921; 799.6707; 366.3736
876,6832	13,67	$C_{52}H_{98}NO_8P$	M-H_2O-H	PE-NMe 24:1/22:2	-2,24	0,002098	Glycerophospholipids	Glycerophosphoethanolamines	859.6921; 168.0423
706,6366	13,77	$C_{43}H_{83}NO_3$	M+FA-H	Cer 43:2;O_2	1,61	0,055778	Sphingolipids	Ceramides	660.6300
738,6622	13,87	$C_{44}H_{87}NO_4$	M+FA-H	Cer 44:1;O_2	0,66	0,004887	Sphingolipids	Ceramides	692.6863
862,7125	13,88	$C_{50}H_{99}NO_8$	M+Na-2H	HexCer 44:0;O_2	0,90	0,000174	Neutral glycosphingolipids	Simple Glc series	803.6985; 801.6866
900,7099	13,92	$C_{50}H_{98}NO_7P$	M+FA-H	PC O-42:2	4,21	0,049704	Glycerophospholipids	Glycerophosphocholines	840.6901; 255.2327
680,6215	13,94	$C_{41}H_{81}NO_3$	M+FA-H	Cer 41:1;O_2	2,63	0,057469	Sphingolipids	Ceramides	634.6155
708,6479	13,97	$C_{44}H_{87}NO_5$	M-H	Cer 18:1;O_3/26:0;O	-4,52	0,004400	Sphingolipids	Ceramides	660.6326; 408.3829; 281.2481
720,6521	14,12	$C_{44}H_{85}NO_3$	M+FA-H	Cer 44:2;O_2	1,47	0,083455	Sphingolipids	Ceramides	674.6469
595,4944	2,44	$C_{35}H_{66}O_4$	M+FA-H	DG P-14/18:1	0,18	0,001768	Glycerolipids	Diradylglycerols	351.2513; 311.2220; 295.2271; 283.2634; 281.2479; 255.2322
717,5200	3,10	$C_{37}H_{73}N_2O_6P$	M+FA-H	SM 32:2;O_2	1,78	0,049754	Sphingolipids	Phosphosphingolipids	657.4981; 168.0424; 78.9584;
1598,0348	3,94	$C_{89}H_{150}O_{17}P_2$	M+FA-H	CL 80:12	1,12	0,002967	Glycerophospholipids	Glycerophosphoglycerophosphoglycerols	787.5240; 303.2323; 293.2636; 255.2325
552,4637	3,98	$C_{32}H_{61}NO_3$	M+FA-H	Cer 14:1;O2/18:1	0,65	0,067776	Sphingolipids	Ceramides	283.2636; 255.2325; 227.2015; 199.1702
743,4690	3,98	$C_{43}H_{69}O_8P$	M-H	PA 22:5/18:3	4,43	0,002850	Glycerophospholipids	Glycerophosphates	662.4835; 483.2509; 465.2408; 415.2252; 330.2516; 329.2483
745,5509	4,06	$C_{39}H_{77}N_2O_6P$	M+FA-H	SM 34:2;O_2	1,03	0,006539	Sphingolipids	Phosphosphingolipids	745.5511; 685.5292; 168.0423; 78.9583
798,5305	4,10	$C_{42}H_{76}NO_8P$	M+FA-H	PC 20:4/14:4	1,89	0,057414	Glycerophospholipids	Glycerophosphocholines	303.2321; 227.2014
716,4662	4,28	$C_{41}H_{70}NO_8P$	M-H_2O-H	PE 14:0/22:6	0,18	0,051209	Glycerophospholipids	Glycerophosphoethanolamines	463.2249; 391.2247; 327.2318; 283.2427; 227.2009; 203.1800
786,5741	4,28	$C_{42}H_{79}NO_9$	M+FA-H	HexCer 36:2;O_3	0,53	0,002154	Neutral glycosphingolipids	Simple Glc series	758.5355; 283.2641; 255.2321; 227.2009; 199.1701; 119.0345
557,4572	4,29	$C_{36}H_{62}O_4$	M-H	FAHFA 36:4	-0,58	0,057539	Fatty Acyls	Fatty acids and conjugates	295.2270; 279.2319
581,4551	4,29	$C_{38}H_{62}O_4$	M-H	FAHFA 38:6	-4,19	0,004838	Fatty Acyls	Fatty acids and conjugates	301.2166; 279.2319
800,5455	4,64	$C_{42}H_{78}NO_8P$	M+FA-H	PC 34:3	1,11	0,002091	Glycerophospholipids	Glycerophosphocholines	740.5248
554,4791	5,10	$C_{32}H_{63}NO_3$	M+FA-H	Cer 32:1;O_2	0,22	0,057239	Sphingolipids	Ceramides	508.4743
645,4515	5,10	$C_{35}H_{67}O_8P$	M-H	PA 16:1/16:0	2,15	0,058109	Glycerophospholipids	Glycerophosphates	409.2353; 391.2244; 255.2321; 152.9952
747,5663	5,21	$C_{39}H_{79}N_2O_6P$	M+FA-H	SM 34:1;O_2	0,69	0,002420	Sphingolipids	Phosphosphingolipids	747.5660; 687.5450; 449.3141; 168.0423
580,4950	5,25	$C_{34}H_{65}NO_3$	M+FA-H	Cer 18:2;O_2/16:0	0,60	0,085827	Sphingolipids	Ceramides	281.2477; 277.2010

1502,0671	5,35	$C_{41}H_{77}O_8P$	2M+FA-H	PA 16:1/22:1	-1,61	0,002165	Glycerophospholipids	Glycerophosphates	671.4666; 391.2245; 281.2477; 255.2320; 253.2166
1676,1093	5,37	$C_{43}H_{83}O_{13}P$	2M-H	PI 16:0/18:0	1,39	0,001990	Glycerophospholipids	Glycerophosphoinositols	788.5249; 255.2320
732,5198	5,53	$C_{39}H_{76}NO_9P$	M-H	PS P-16:0/17:0	1,83	0,051244	Glycerophospholipids	Glycerophosphoserines	437.2665; 419.2555
746,5139	5,93	$C_{43}H_{74}NO_7P$	M-H	PE O-16:0/22:6	1,20	0,049707	Glycerophospholipids	Glycerophosphoethanolamines	436.2824; 418.2707; 417.2406; 327.2319; 239.2384; 229.1960
419,2561	6,00	$C_{21}H_{43}O_7P$	M-H_2O-H	LPA 18:0/0:0	-1,50	0,070838	Glycerophospholipids	Glycerophosphates	283.2633; 171.0060; 152.9951; 134.9847; 96.9689; 78.9583
721,5032	6,00	$C_{37}H_{73}O_8P$	M+FA-H	PA 18:0/16:0	0,99	0,051228	Glycerophospholipids	Glycerophosphates	437.2662; 283.2633; 96.9689;
723,4986	6,00	$C_{41}H_{73}O_8P$	M-H	PA 20:4/18:0	2,20	0,003608	Glycerophospholipids	Glycerophosphates	457.2357; 439.2243; 419.2556; 283.2633; 96.9689; 78.9583
810,5305	6,00	$C_{43}H_{76}NO_8P$	M+FA-H	PE 24:4/18:1	1,82	0,000175	Glycerophospholipids	Glycerophosphoethanolamines	836.5458; 749.5134; 419.2556; 305.2468; 303.2319; 283.2633
781,5045	6,00	$C_{42}H_{73}O_8P$	M+FA-H	PA 20:4/19:1	2,76	0,002378	Glycerophospholipids	Glycerophosphates	457.2357; 419.2556; 303.2319; 283.2633; 259.2427; 96.9689
1548,0302	6,06	$C_{42}H_{74}NO_8P$	2M+FA-H	PE-NMe 36:5	1,03	0,054096	Glycerophospholipids	Glycerophosphoethanolamines	782.4990; 305.2473; 255.2321; 253.2172; 241.2168; 168.0425
714,5083	6,07	$C_{39}H_{74}NO_8P$	M-H	PE 18:2/16:0	0,52	0,006456	Glycerophospholipids	Glycerophosphoethanolamines	458.2631; 452.2777; 279.2319; 255.2321; 253.2169; 140.0112
768,5560	6,07	$C_{43}H_{80}NO_8P$	M-H	PE 38:3	1,47	0,000229	Glycerophospholipids	Glycerophosphoethanolamines	714.5082
782,4995	6,09	$C_{41}H_{72}NO_8P$	M+FA-H	PE 18:2/18:3	2,39	0,062799	Glycerophospholipids	Glycerophosphoethanolamines	281.2473; 279.2319; 140.0112
744,5209	6,11	$C_{39}H_{74}NO_7P$	M+FA-H	PE O-16:0/18:2	3,39	0,002437	Glycerophospholipids	Glycerophosphoethanolamines	478.2930; 476.2778; 452.2780; 281.2474; 279.2319; 255.2321
1603,9960	6,46	$C_{95}H_{148}O_{17}P_2$	M-H_2O-H	CL 86:19	-3,29	0,002406	Glycerophospholipids	Glycerophosphoglycerophosphoglycerols	303.2320; 259.2426; 231.2116; 205.1957
719,4891	6,73	$C_{38}H_{73}O_{10}P$	M-H	PG 18:0/14:1	3,09	0,051336	Glycerophospholipids	Glycerophosphonylglycerol	283.2632
775,5497	6,81	$C_{41}H_{79}O_8P$	M+FA-H	PA 20:0/18:1	0,35	0,002500	Glycerophospholipids	Glycerophosphates	281.2481; 255.2323
582,5101	6,83	$C_{34}H_{67}NO_3$	M+FA-H	Cer 18:1;O_2/16:0	-0,36	0,049759	Sphingolipids	Ceramides	536.5042; 506.4942; 280.2641; 263.2388; 254.2492; 237.2218
748,5296	6,83	$C_{39}H_{78}NO_7P$	M+FA-H	PE O-34:1	-1,19	0,000105	Glycerophospholipids	Glycerophosphoethanolamines	724.5269; 462.2983; 438.2980; 259.2426; 205.1960; 89.0236
816,5204	6,85	$C_{46}H_{78}NO_{10}P$	M-H_2O-H	PS 20:4/20:2	2,34	0,000119	Glycerophospholipids	Glycerophosphoserines	303.2319; 259.2426; 205.1958
608,5264	7,02	$C_{36}H_{69}NO_3$	M+FA-H	Cer 18:2;O_2/18:0	0,81	0,057405	Sphingolipids	Ceramides	333.2702; 281.2476; 255.2321
742,5404	7,02	$C_{41}H_{78}NO_8P$	M-H	PE 18:2/18:0	1,61	0,003962	Glycerophospholipids	Glycerophosphoethanolamines	480.3089; 281.2476; 140.0112
744,5555	7,09	$C_{41}H_{80}NO_8P$	M-H	PE 18:1/18:0	0,89	0,000102	Glycerophospholipids	Glycerophosphoethanolamines	480.3088; 464.3140; 281.2477; 140.0112
880,6087	7,19	$C_{48}H_{86}NO_8P$	M+FA-H	PE-NMe 20:4/22:1	1,69	0,055956	Glycerophospholipids	Glycerophosphoethanolamines	820.5845; 794.5714; 303.2319; 259.2427
913,5835	7,20	$C_{49}H_{87}O_{13}P$	M-H	PI 22:4/18:0	2,60	0,000127	Glycerophospholipids	Glycerophosphoinositols	329.2477; 285.2593
887,5676	7,31	$C_{47}H_{85}NO_{13}P$	M-H	PI 22:3/16:0	2,36	0,057372	Glycerophospholipids	Glycerophosphoinositols	860.5439; 751.5292; 594.2823; 331.2630

837,5504	7,60	$C_{43}H_{83}O_{13}P$	M-H	PI 16:0/18:0	0,62	0,055699	Glycerophospholipids	Glycerophosphoinositols	281.2476; 255.2321
1524,0278	7,64	$C_{41}H_{74}NO_8P$	2M+FA-H	PE 18:1/18:3	-0,53	0,001983	Glycerophospholipids	Glycerophosphoethanolamines	766.5401; 281.2477; 255.2321; 205.1960; 140.0113
856,6083	8,06	$C_{46}H_{86}NO_8P$	M+FA-H	PC 18:3/20:0	1,25	0,051531	Glycerophospholipids	Glycerophosphocholines	796.5873; 742.5399; 279.2320
1327,8150	8,40	$C_{64}H_{118}N_2O_{23}$	M+FA-H	HexNAc-Cer 38:1;O_2	3,36	0,049698	Sphingolipids	Neutral glycosphingolipids	1281.8087; 840.6150; 780.5914
674,5149	8,59	$C_{37}H_{74}NO_7P$	M-H	PE O-32:1	2,86	0,061801	Glycerophospholipids	Glycerophosphoethanolamines	480.3094; 406.2804; 405.2761
700,5291	8,72	$C_{39}H_{78}NO_8P$	M-H_2O-H	PE 16:0/18:0	0,54	0,003134	Glycerophospholipids	Glycerophosphoethanolamines	464.3136; 446.3031; 436.2827; 418.2721; 267.2686; 140.0113
776,5608	8,72	$C_{45}H_{80}NO_7P$	M-H	PE O-22:5/18:0	1,09	0,000108	Glycerophospholipids	Glycerophosphoethanolamines	464.3136; 446.3031; 403.2614; 329.2475; 285.2583; 267.2686; 140.0113
610,5418	9,21	$C_{36}H_{71}NO_3$	M+FA-H	Cer 18:1/18:0;O_2	0,33	0,004218	Sphingolipids	Ceramides	564.5366; 308.2957; 255.2322
735,5369	9,37	$C_{43}H_{77}O_7P$	M-H	PA P-18:1/22:4	4,73	0,049694	Glycerophospholipids	Glycerophosphates	331.2633; 78.9583
636,5579	9,44	$C_{38}H_{73}NO_3$	M+FA-H	Cer 18:2/20:0;O_2	1,12	0,055918	Sphingolipids	Ceramides	283.2630; 281.2477
772,5873	9,46	$C_{43}H_{84}NO_8P$	M-H	PE-NMe2 18:1/18:0	1,46	0,075295	Glycerophospholipids	Glycerophosphoethanolamines	508.3408; 283.2629; 281.2477; 168.0424
832,6086	9,46	$C_{44}H_{86}NO_8P$	M+FA-H	PC 36:1	1,69	0,064319	Glycerophospholipids	Glycerophosphocholines	772.5871; 508.3405; 224.0688
900,5970	9,46	$C_{47}H_{86}NO_{10}P$	M+FA-H	PS 22:2/19:1	-0,15	0,063071	Glycerophospholipids	Glycerophosphoserines	283.2629; 281.2477
662,5738	9,47	$C_{40}H_{75}NO_3$	M+FA-H	Cer 40:3;O_2	1,42	0,067942	Sphingolipids	Ceramides	616.5674
688,5894	9,61	$C_{42}H_{77}NO_3$	M+FA-H	Cer 42:4;O_2	1,36	0,070795	Sphingolipids	Ceramides	642.5839; 388.3582
829,6455	9,61	$C_{45}H_{89}N_2O_6P$	M+FA-H	SM 40:2;O_2	1,82	0,003057	Sphingolipids	Phosphosphingolipids	769.6235
844,5494	9,67	$C_{46}H_{82}NO_9P$	M+Na-2H	PS O-40:5	2,47	0,002785	Glycerophospholipids	Glycerophosphoserines	776.5612; 231.2118
